# Targeted single molecule sequencing methodology for ovarian hyperstimulation syndrome

**DOI:** 10.1186/s12864-015-1451-2

**Published:** 2015-04-03

**Authors:** Funda Orkunoglu-Suer, Arthur F Harralson, David Frankfurter, Paul Gindoff, Travis J O’Brien

**Affiliations:** Department of Integrated System Biology, The George Washington University Medical Center, Washington, DC 20037 USA; Department of Pharmacogenomics, Bernard J. Dunn School of Pharmacy, Shenandoah University, Ashburn, VA USA; Department of Obstetrics and Gynecology, The George Washington University Medical Center, Washington, DC 20037 USA; Department of Pharmacology and Physiology, The George Washington University Medical Center, Washington, DC 20037

**Keywords:** Single-molecule sequencing, Droplet-based PCR, Emulsion PCR, Next generation DNA sequencing, Ovarian hyperstimulation syndrome, OHSS

## Abstract

**Background:**

One of the most significant issues surrounding next generation sequencing is the cost and the difficulty assembling short read lengths. Targeted capture enrichment of longer fragments using single molecule sequencing (SMS) is expected to improve both sequence assembly and base-call accuracy but, at present, there are very few examples of successful application of these technologic advances in translational research and clinical testing. We developed a targeted single molecule sequencing (T-SMS) panel for genes implicated in ovarian response to controlled ovarian hyperstimulation (COH) for infertility.

**Results:**

Target enrichment was carried out using droplet-base multiplex polymerase chain reaction (PCR) technology (RainDance®) designed to yield amplicons averaging 1 kb fragment size from candidate 44 loci (99.8% unique base-pair coverage). The total targeted sequence was 3.18 Mb per sample. SMS was carried out using single molecule, real-time DNA sequencing (SMRT® Pacific Biosciences®), average raw read length = 1178 nucleotides, 5% of the amplicons >6000 nucleotides). After filtering with circular consensus (CCS) reads, the mean read length was 3200 nucleotides (97% CCS accuracy). Primary data analyses, alignment and filtering utilized the Pacific Biosciences® SMRT portal. Secondary analysis was conducted using the Genome Analysis Toolkit for SNP discovery l and wANNOVAR for functional analysis of variants. Filtered functional variants 18 of 19 (94.7%) were further confirmed using conventional Sanger sequencing. CCS reads were able to accurately detect zygosity. Coverage within GC rich regions (i.e.*VEGFR*; 72% GC rich) was achieved by capturing long genomic DNA (gDNA) fragments and reading into regions that flank the capture regions. As proof of concept, a non-synonymous *LHCGR* variant captured in two severe OHSS cases, and verified by conventional sequencing.

**Conclusions:**

Combining emulsion PCR-generated 1 kb amplicons and SMRT DNA sequencing permitted greater depth of coverage for T-SMS and facilitated easier sequence assembly. To the best of our knowledge, this is the first report combining emulsion PCR and T-SMS for long reads using human DNA samples, and NGS panel designed for biomarker discovery in OHSS.

**Electronic supplementary material:**

The online version of this article (doi:10.1186/s12864-015-1451-2) contains supplementary material, which is available to authorized users.

## Background

Sequence capture enrichment strategies and single molecule sequencing (SMS) are expected to increase the rate of gene discovery for genetically heterogeneous diseases. There have been several recent reports on the successful application of SMS to interrogate both viral [1-4] and bacterial [3,5-10] genomes. At present, there are very few examples of successful application of these technologic advances in translational research and clinical testing in humans. Recently, the targeted exon sequencing of 238 cancer gene mutations from tumor/blood samples using the PacBio RS platform was reported indicating that achieving longer reads with SMS was feasible in human samples [11]. In that study, target enrichment was achieved through the generation of amplicons averaging 340 bp in length.

More than one million couples worldwide seek reproductive assistance each year because of infertility [12]. Unfortunately, infertility therapy involving controlled ovarian stimulation (COH) may result in potentially fatal iatrogenic ovarian hyperstimulation syndrome (OHSS). OHSS reported as leading cause of maternal mortality in UK [13]. The overarching objective of this study was to identify predictive genetic biomarkers for outcome to controlled ovarian hyperstimulation (COH). Patient response to COH is variable and likely influenced by a diverse array of genetic (and epigenetic) factors requiring sophisticated next-generation sequencing (NGS) techniques for elucidation. To date, there have been no tools developed to query all regions (including intronic and 5′ and 3′UTR flanking sequences) of candidate genes for COH and its major iatrogenic complication, ovarian hyperstimulation syndrome (OHSS). We have developed a targeted SMS (T-SMS) panel containing 44 loci that have been implicated in either response to COH or OHSS. Our approach utilized droplet-based emulsion PCR for the generation of 1913 amplicons averaging 1 kb in length for T-SMS. We report the successful development and implementation of this novel technique and an offer proof of concept of its utility.

## Methods

### Sample collection and processing

This study was approved by George Washington University Institutional Review Board. It was open to all adult (>18 years of age) female patients recruited previously treated or currently seeking OHSS treatment at the GW Fertility and IVF Center. Written informed consent for participation was obtained from the participants prior to sample collection. Wet lab and dry lab work carried out within Genetics in Medicine Research Institute of Children’s National Medical Center. Ovarian hyperstimulation syndrome, non-responders and hyper-responders to ovarian simulation were defined clinically based on the criteria established by Navot [14,15]. Approximately 5 mL of blood was collected for genetic analysis.

Total genomic DNA was extracted from 200 uL EDTA anti-coagulated venous blood using magnetic beads (Maxwell® 16 DNA Kit DNA Purification Kit (Promega, US) on a fully automated system. DNA (1 uL) quality and quantity was measured (260/280 and 260/230 ratio) using a Qubit dsDNA HS assay kit and system (Invitrogen, US). Samples showing RNA contamination (260/280 ratio >2) were discarded and samples with a 260/230 ratio less than one were excluded from further processing. DNA was quantified in duplicate for each sample and the mean value was used for further calculations. DNA integrity (1 uL) was analyzed using an E-Gel® Agarose Gel Electrophoresis System on a 0.8% agarose gel. Samples showing fragmentation were discarded and re-isolated from fresh patient samples. DNA (3 mg) was sonicated using a Covaris S220 system (Covaris, US) for 180 s to ~5 kb fragment size (20% duty cycle, 5 intensity, 200 cycles per burst).

### Target gene list and primer design

The target gene list was developed from an extensive literature search. It included genes that were a) implicated in COH response, b) associated with OHSS or c) regulated gonadotropin action and/or ovarian angiogenesis. Genes included in the target list either a) harbored variants associated with COH outcome, b) displayed differential expression (mRNA, protein) in OHSS and/or 3) played a significant role in gonadotropin signaling or in regulating vascular permeability in the ovary. The Genomic targets were comprehensive and included intronic, exonic, 5′ and 3′ untranslated regions (UTRs) of the target genes. The total targeted sequence consisted of 3.18 Mb covering 44 loci with 1X tiling (see Additional file 1). The primer library was designed using the manufacturer’s parameters (Rain Dance Technologies, US) and all primers were first tested with Primer3 (http://frodo.wi.mit.edu/primer3/). The primer design pipeline performed an exhaustive primer selection across all of the regions submitted and generated 1951 unique amplicons (average amplicon length ~1 kb) using 3756 primers (T_m_ = 55-65°C, 99.8% success rate). Repeat masking was not performed on the input regions to the primer design pipeline. Primers were designed to provide ~100 bp overlap between adjacent amplicons and avoided primer binding to SNPs and repeat regions. There was no allele dropouts discovered in the final design.

### Droplet-Based multiplex amplification

Amplification was carried out similar to previously described methods [16] and according to the manufacturer’s protocol. Following amplification each PCR emulsion was broken to release individual amplicons from the PCR droplets and samples were purified using a MinElute column (Qiagen, US) following the manufacturer’s recommended protocol. Purified amplicons were then tested on an Agilent Bioanalyzer (Agilent, US) and Qubit (Qiagen, US) to assure quality and quantity and confirm that the amplicon profile matches the expected histogram profile.

### Library construction and single molecule, Real-Time sequencing (SMRT)

Amplicons (1 μg) were converted to SMRTbell™ templates using the PacBio® RS DNA Template Preparation Kit (catalog #001-322-716), incubated for 15 min at 25°C and further purified with a 0.6X AMPure XP clean-up kit and eluted in 30 μl buffer. Blunt adapters were ligated to each amplicon to facilitate circle replication [SMRTbell™ template sequencing] and to permit error control by calculating the consensus (‘circular consensus sequence’ or CCS). Exonuclease incubation was carried out in order to remove all unligated adapters. Samples were extracted twice (0.6X AMPure beads) and the final “SMRT bells” were eluted in 10 μl EB. Final quantification was carried out on an Agilent 2100 Bioanalyzer with 1 μl of library. The amount of primer and polymerase required for the binding reaction was determined using the SMRT bell concentration (ng/μl) and insert size previously determined using the manufacturer-provided calculator. Primers were annealed and polymerase was bound using the DNA/Polymerase Binding Kit 1.0 (PacBio catalog #001-359-802). The complexes were stored at −20°C or diluted for immediate sequencing.

Sequencing mixes were diluted to the required concentration with the manufacturer provided dilution buffer prior to loading onto 96-well plates. Sample plates were loaded onto the instrument along with the DNA Sequencing Kit 1.0 (catalog #001-379-044). Sequencing was performed using PacBio SMRT sequencing technology as previously described (4) using a SMRT Cell 8Pac. In all sequencing runs, 2 x 45 min movies were captured for each SMRT Cell loaded with a single binding complex.

### Data analyses

The data analyses pipeline is provided in Table 1. Primary filtering analysis was first performed using the PacBio *RS* server prior to data being transferred to the SMRT Portal using the SMRT analysis pipeline version 1.3.3 (http://www.smrtcommunity.com/SMRT-Analysis/Software/SMRT-Pipe); http://www.pacificbiosciences.com/products/pacificbio-rs-workflow-main/). Secondary analysis was conducted using the Genome Analysis Toolkit (GATK) (http://www.broadinstitute.org/gatk/) embedded in the SMRT Portal. Output files (VCF and BAM files) were transferred to wANNOVAR (http://wannovar.usc.edu/) for variant (SNP) calling (relative to reference sequence assembly; hg19). The project was registered with the NIH bioproject database (http://www.ncbi.nlm.nih.gov/bioproject/193545). All sequence data was made accessible from the NIH next generation sequence read archive (SRA) data base (http://www.ncbi.nlm.nih.gov/Traces/sra).

**Table 1:** **Data pipeline**

**Primary Analyses**	**Q metrics**	**SMRT standard pipeline**
**Secondary Analyses**	BLASR de novo CCS aligner algorithm filter v1 (hg19)	BLASR de novo CCS aligner algorithm was used for SNP calling using CCS reads. Reads were filtered by length/quality and mapped to reference sequence (UCSC, hg19). Base quality scores were recalibrated, and consensus Filter v1: Min Read Length bp: 50, Minimum Sub Read Length 50
**GATK**	Overview	Variants identified using the GATK Unified Genotyper for Bayesian diploid and haploid SNP calling using base quality score recalibration and default settings. Indel calling was not included in SMRT pipeline
**ANNOVAR**		Functional annotation of variants
**Data visualization**	Overview	SMART view, UCSC Genome browser, R circos plot, Partek

### Sanger validation of variants

Validation of SMS variants was conducted by Sanger DNA sequencing as previously described [17]. Primers were designed using the National Center for Biotechnology Information website (http://www.ncbi.nlm.nih.gov) and University of Santa Cruz Genome Browser (https://genome.ucsc.edu). Multiple sequence alignments were carried out using ChromasPro software (Technelysium Pty Ltd). All variants were reported according to standard nomenclature. (http://www.hgvs.org/mutnomen/)

## Results and discussion

### Single molecule sequencing of DNA libraries

We targeted the entire coding region (exons/introns) and the 3′ and 5′ UTR non-coding sequences of 44 candidate loci covering ~3.18 Mb per sample. Our primer design yielded 3756 primer pairs that generated 1951 amplicons that were confirmed to be 1 kb in length (not shown). Amplicons were tiled to have an average overlap of 100 base pairs (bp) to facilitate coverage and assembly. For the SMS-generated raw reads the average read length was 1178 nucleotides (nt) and ~5% were >6000 nt. SMS (2 chips per sample) was successful in capturing 100% sequence information from 1816 out of the 1951 amplicons targeted (93.1%). After filtering for circular consensus (CCS) reads, the mean read length was 3200 nt which was likely due to the use of a longer sequencing protocol to accommodate the larger size (1 kb) of the amplicons (Table 2). The mean mapped CCS read accuracy was 97%. A small percentage (5%) of consensus reads of were >6215 nt.

**Table 2:** **Characteristics of captured sequence**

**Characteristic**	**Result**
Sequence yield per run (pre filter base)	800 Mb
Sequence run per sample	2 chips
Run time	2 movies, 45 min each
Mean Accuracy	10X CCS, 97.3%
Targeted Accuracy	10 X CCS, 100%
Mean Read length	3200 nt
Mean mapped read length	900 bp
Insert size	1 kb
DNA requirements	500 ng/uL

We generated average 900 bp mean mapped subreads with a mean zero-mode waveguide (ZMW) occupancy of 85%. In our primary design, we calculated target coverage depth using the manufacturer’s formula (6). Based on this, we used 2 chips per sample and SMS data were collected in 2 x 45-min movies to attain 17X targeted CCS coverage depth. These results are in agreement with recent targeted sequencing studies shown to have higher coverage depth than exome and whole genome sequencing using different enrichment strategies [18]. Previous work has suggested that 10X CCS coverage depth (or 50X single read coverage = QV50) can accurately (100%) distinguish between heterozygous and homozygous SNPs [17]. Consequently, our filtering was set to a minimum cut-off of 10 X CCS coverage depth.

### Repetitive sequences

**Figure 1:**
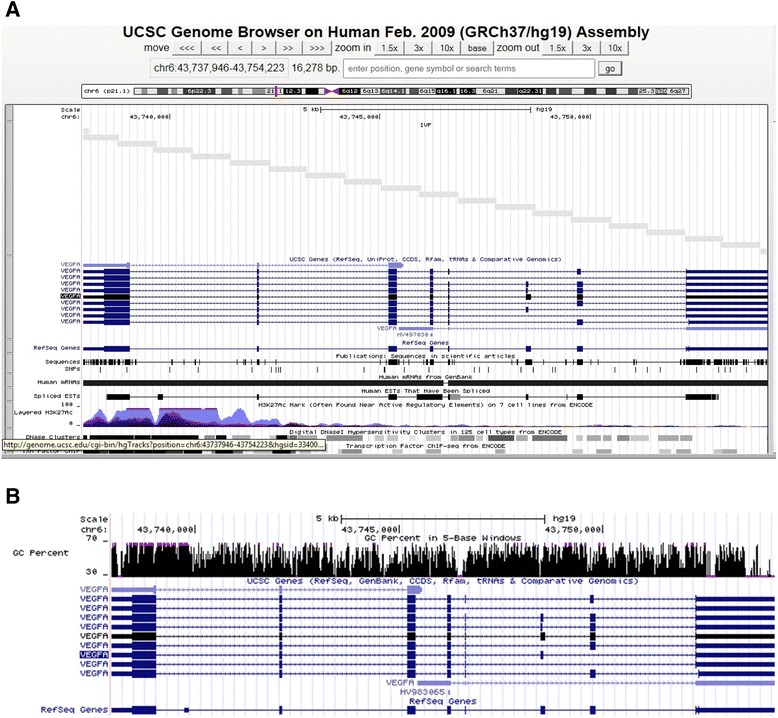
**Read length vs. GC coverage.** This is a representative example of typical read length and coverage **(A)** of GC rich regions (*VEGFA* gene) and sequence results imported as custom track in UCSC Genome Browser. Fragments (1 kb) were tiled to cover the entire genomic region of the *VEGFA* gene (grey bars above sequence). There were a total of 42 amplicons (intragenic sequences totaled 34,268 bp) with 50–150 bp overlap. **B**. Screen capture showing the high GC content (72%) of the *VEGFA* gene which was successfully 005B1:1] sequenced.

**Figure 2:**
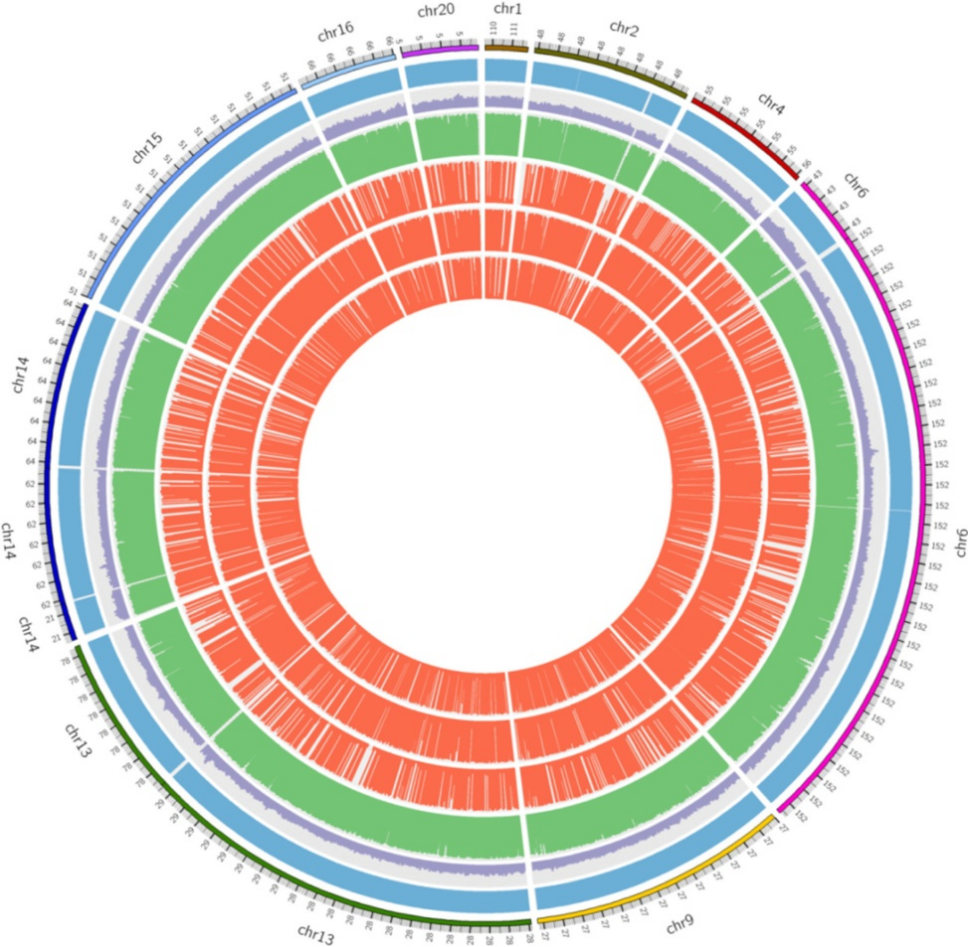
**Uniform Coverage between amplicons using Droplet PCR with SMS technology.** Circos plot illustrating the relative coverage of target sequences in 3 samples. Outermost blue displays target genomic sequence with respect to chromosomal location. For each target sequence, the percentage GC content is provided in purple (scale 0–100). The bases covered for bait regions are shown in green (scale 1–1000). The coverage of SMS for 3 representative samples is provided in red.

Guanine-cytosine (GC)-rich regions of the genome pose significant challenges for high-throughput DNA sequencing. We were able to sequence 1 kb amplicons generated from GC-rich regions in our targeted sequences. Moreover, we were able to align these GC-rich sequences with similar success as non-GC-rich regions which is consistent with other reports [6]. As proof of concept, Figure 1 shows tiled coverage of the *VEGFA* gene using total 42 amplicons (Figure 1A). *VEGFA* has ~72% GC content (Figure 1B). The uniform coverage for three representative samples aligned against the hg19 reference sequence is provided in the circos plot in Figure 2.

### Confirmation of T-SMS base calls

**Figure 3:**
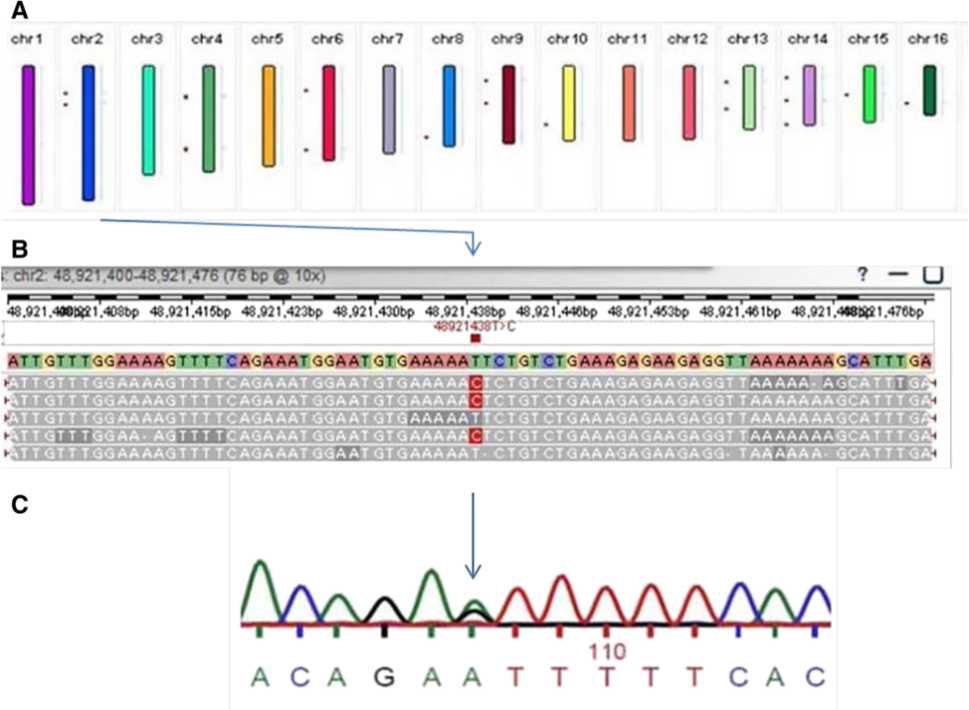
**Validation of rs12470652 identified by T-SMS.** SMRT® View screenshot of secondary data analysis from SMRT® Portal (A and B). Validation of the *LHCGR* rs12470652 heterozygous variant discovered using T-SMS by Sanger DNA sequencing. The figure depicts the process of biomarker identification by SMS **(A** and **B)** and validation by conventional Sanger sequencing **(C)**.

Base-call accuracy is of great concern with next generation sequencing technologies. We have validated 19 SNPs identified by T-SMS with Sanger DNA sequencing. For those exonic variants identified by T-SMS, 18 of 19 (94.7%) of the SNPs were verified by Sanger DNA sequencing. As an example, Figure 3 shows the validation of rs12470652 in the luteinizing hormone choriogonadotropin receptor (*LHCGR*) gene. This missense variant (NP_000224.2:p. Asn291Ser; T > C) passed all the filters and variant annotation analyses steps (Figure 3A) and was further validated using conventional Sanger sequencing (Figure 3B) and here we report it in two severe OHSS patients.

## Conclusions

Targeted sequencing approaches are advantageous for enriching variant identification, simplifying data analysis and avoiding ethical issues surrounding incidental findings. We have developed a custom protocol and data processing pipeline for generating 1 kb amplicons by emulsion PCR for T-SMS. Our preliminary analysis has initially focused on coding region variants in each of our 44 candidate loci for OHSS. Although smaller amplicons may theoretically yield more readings than larger fragments, we have found that fixed/same size and longer amplicons work effectively with droplet PCR enrichment combined with SMS. Employing T-SMS technology has provided improved resolution by yielding longer reads and sequencing many target genes in a relatively short period of time (45 min). Moreover, T-SMS of large amplicons had low composition bias and an error profile that is orthogonal to other next generation sequencing platforms that have promise for clinical diagnosis. To the best of our knowledge, this is the first study reporting the successful sequencing of 1 kb amplicons utilizing droplet PCR combined with SMS technology from human samples. These data show excellent promise for follow-up studies with a larger number of OHSS cases.

### Availability of supporting data

Supporting data is included as additional files. Project details and Sequence Data registered and can be found in the publicly available databases: http://www.ncbi.nlm.nih.gov/bioproject/193545http://www.ncbi.nlm.nih.gov/pmc/articles/PMC2808927/.

## Additional file

Additional file 1:
**Targeted gene list.**

## Abbreviations

SMS: Single molecule sequencing
COH: Controlled ovarian hyperstimulation
OHSS: Ovarian hyperstimulation syndrome
NGS: Next generation DNA sequencing
PCR: Polymerase chain reaction
GDNA: Genomic DNA
CCS: Circular Consensus Sequencing
SMRT®: Single molecule, real-time
T-SMS: Targeted SMS
ZMW: Zero-mode waveguide
SNP: Single nucleotide polymorphism
