# Supplementary material for: Targeted single molecule sequencing methodology for ovarian hyperstimulation syndrome
Source: BMC Genomics. 2015 Apr 3;16(1):264. doi: 10.1186/s12864-015-1451-2 (PMC4397691; doi:10.1186/s12864-015-1451-2)
Supplement: Additional file 1: — Targeted gene list. [file 12864_2015_1451_MOESM1_ESM.docx]

**Additional File 1: Targeted gene list**

| **Gene ID** | **RefSeq_ID** | **Chromosome** |
| --- | --- | --- |
| *VEGFA* | NG_008732.1 | 6 |
| *PROK1* | NP_115790.1 | 1 |
| *PROKR1* | NP_620414 | 2 |
| *PROKR2* | NG_008132 | 20 |
| *FLT1* | NG_012003 | 13 |
| *KDR* | NG_012004 | 4 |
| *HIF1A* | NM_001530 | 14 |
| *LHCGR* | NG_008193 | 2 |
| *FSHR* | NG_008146 | 2 |
| *CGA* | NM_000735 | 6 |
| *CGB* | NM_000737 | 19 |
| *TSHB* | NM_000549 | 1 |
| *CDH5* | NM_001795 | 16 |
| *OCLN* | NM_002538 | 5 |
| *ESR1* | NM_001122742 | 6 |
| *ESR2* | NM_001040275 | 14 |
| *INHBA* | NM_002192 | 7 |
| *INHA* | NM_002191 | 2 |
| *INHBB* | NM_002193 | 2 |
| *INHBC* | NM_005538 | 12 |
| *CLDN5* | NM_001130861 | 22 |
| *ACVR2A* | NM_001616 | 2 |
| *ACVR2B* | NM_001106 | 3 |
| *ACVR1* | NM_001111067 | 2 |
| *ACVR1B* | NM_004302 | 12 |
| *LPAR1* | NM_057159 | 9 |
| *LPAR2* | NM_004720 | 19 |
| *LPAR3* | NM_012152 | 1 |
| *LPAR4* | NM_005296 | X |
| *THBS1* | NM_003246 | 15 |
| *FST* | NM_006350 | 5 |
| *ANG* | NM_001145 | 14 |
| *EDN1* | NM_001955 | 6 |
| *EDN2* | NM_001956 | 1 |
| *EDNRB* | NM_000115 | 13 |
| *EDNRA* | NM_001957 | 4 |
| *IL8* | NM_000584 | 4 |
| *IL8RA* | NM_000634 | 2 |
| *FGF2* | NM_002006 | 4 |
| *CYP19A1* | NM_000103 | 15 |
| *ANGPT1* | NM_001146 | 8 |
| *ANGPT2* | NM_001118887 | 8 |
| *TEK* | NM_000459 | 9 |
